# Supplementary material for: Examining intentions to take iron supplements to inform a behavioral intervention: The Reduction in Anemia through Normative Innovations (RANI) project
Source: PLoS One. 2021 May 11;16(5):e0249646. doi: 10.1371/journal.pone.0249646 (PMC8112683; doi:10.1371/journal.pone.0249646)
Supplement: S1 Appendix — (DOCX) [file pone.0249646.s001.docx]

**Reduction in Anemia through Normative Innovations (RANI) Project Baseline Questionnaire**

**(Selected Items)**

This work was supported by a grant from The Bill and Melinda Gates Foundation (OPP1182519) to the George Washington University, Rajiv N. Rimal, principal investigator. The funders had no role in study design, data collection and analysis, decision to publish, or preparation of the manuscript.

| Pregnancy Statusଗର୍ଭାବସ୍ଥାର ସ୍ଥିତି | | |
| --- | --- | --- |
| **Directions: Say,** “Today I’m going to ask you about anemia. You may have heard it referred to as ‘lack of blood.’ I will also ask you about iron-folic tablets, also known as IRON BATIKA(tablets). ” **Show the participant the IRON BATIKA(tablet) and say,** “This is what they look like. Perhaps you have seen them before.”**ନିର୍ଦେଶ**: ଉତ୍ତରଦାତାଙ୍କୁ କୁହନ୍ତୁ: “ଆଜି ମୁଁ ଆପଣଙ୍କୁ ଆନେମିଆ ବିଷୟରେ ପଚାରିବାକୁ ଯାଉଛି \| ଏହାକୁ ରକ୍ତହୀନତା ମଧ୍ୟ କୁହାଯାଏ ବୋଲି ଆପଣ ଶୁଣିଥାଇ ପାରନ୍ତି \|ମୁଁ ଆପଣଙ୍କୁ ଆଇରନ ଫୋଲିକ ବଟିକା ବିଷୟରେ ମଧ୍ୟ ପଚାରିବି, ଯାହା ଆଇରନ ବଟିକା ବୋଲି ମଧ୍ୟ ଜଣାଯାଏ \|”ଅଂଶଗ୍ରହଣକାରୀଙ୍କୁ ଆଇରନବଟିକାଦେଖାଇ କୁହନ୍ତୁଯେ, “ଏହି ବଟିକା ଗୁଡିକଏହିପରି ଦେଖାଯାଏ \| ହୁଏତ ଏହାକୁ ଆପଣ ପୂର୍ବରୁ ଦେଖିଥାଇ ପାରନ୍ତି \|”  **Say** “Before I ask you about anemia and IRON BATIKA, I’d first like to ask you…”ଉତ୍ତରଦାତାଙ୍କୁ କୁହନ୍ତୁ: “ମୁଁ ଆପଣଙ୍କୁ ଆନେମିଆ ଏବଂ ଆଇରନ ବଟିକା ବିଷୟରେ ପଚାରିବାପୂର୍ବରୁ, ମୁଁ ଆପଣଙ୍କୁପ୍ରଥମେକିଛିପ୍ରଶ୍ନପଚାରିବାକୁଚାହୁଁଛି ……” | | |
|  | What is your age in years?  ଆପଣଙ୍କ ବୟସ କେତେ (ବର୍ଷରେ) ? | Age/ବୟସ: ________________ (years)  Unknown/ଜଣାନାହିଁ: 999 |
|  | Are you currently single, married, separated, divorced, Widowed?  ଆପଣ ବର୍ତ୍ତମାନ ଏକୁଟିଆ, ବିବାହିତ, ଅଲଗା, ପରିତ୍ୟକ୍ତା, ବିଧବା କି ? | Single/ଏକୁଟିଆ …………1  Married/ବିବାହିତ……2  Separated/ଅଲଗା …… 3  Divorced / ପରିତ୍ୟକ୍ତା …….4  Widowed/ ବିଧବା …………5 |
|  | Are you currently pregnant?  ବର୍ତ୍ତମାନ ଆପଣ ଗର୍ଭବତୀ ଅଛନ୍ତି କି ? | Yes/ହଁ …………………… 1  No/ନାହିଁ………………....0 🡺Preg5  Don’t know………..🡺 Preg5 |
|  | How long have you been pregnant?  ଆପଣ କେତେ ସପ୍ତାହ ବା ମାସ ହେଲା ଗର୍ଭବତୀ ଅଛନ୍ତି? | ______ weeks ସପ୍ତାହସଂଖ୍ୟା  _______ monthsମାସ ସଂଖ୍ୟା |
| Preg4_week_month | you been pregnant in ______ weeks&_______ months  ଆପଣ ______ weeks&_______ months ହେଲା ଗର୍ଭବତୀ ଅଛନ୍ତି? |  |
|  | Are you currently breastfeeding?  ଆପଣ ସ୍ତନ୍ୟପାନ କରାଉଛନ୍ତି କି ? | Yes/ହଁ ………………………… 1  No/ନାହିଁ………………....0 |

| Iron Batika Use ଆଇଏଫ୍ଏ ବଟିକା ବ୍ୟବହାର | | |
| --- | --- | --- |
|  | Have you ever eaten/taken an iron batika (tablet)? (Interviewer should hold up the packet of tablets for the interviewee to see)  ଆପଣ କେବେ ବି ଆଇରନ ବଟିକା ଖାଇଛନ୍ତି କି ?  (ପ୍ରଶ୍ନକକର୍ତ୍ତା ଉତ୍ତରଦାତାଙ୍କୁ ଆଇରନ ବଟିକା ପ୍ୟାକେଟ ଦେଖାଇ ଏହି ପ୍ରଶ୍ନ ପଚାରନ୍ତୁ) | Yes, I am currently taking it/ ହଁ, ମୁଁ ବର୍ତ୍ତମାନ ଖାଉଛି.............1  Yes, I took in the past, but not currently / ହଁ, ମୁଁ ଅତୀତରେ ଖାଇଥିଲି, କିନ୍ତୁ ବର୍ତ୍ତମାନ ଖାଉ ନାହିଁ.............2🡪IFA3  No, I have never taken it / ନା, ମୁଁ କେବେ ବି ଖାଇନାହିଁ\|……………0🡺IFA3 |
|  | How many iron batika did you take in the past seven days?  ଗତ 7 ଦିନରେ ଆପଣ କେତୋଟି ଆଇରନ ବଟିକା ଖାଇଥିଲେ ?  (Interviewer should hold up the packet of tablets for the interviewee to see)  (ପ୍ରଶ୍ନକକର୍ତ୍ତା ଉତ୍ତରଦାତାଙ୍କୁ ଆଇରନ ବଟିକା ପ୍ୟାକେଟ ଦେଖାଇ ଏହି ପ୍ରଶ୍ନ ପଚାରନ୍ତୁ) | **record response**  **ଖାଇଥିବା** ବଟିକା**ସଂଖ୍ୟାକୁ ଲେଖନ୍ତୁ**  If 7 or more, ଯଦି ୭କିମ୍ବା ଏଥିରୁ ଅଧିକା,🡺RP1 |
|  | **Directions: Ask the participant:**  ନିର୍ଦ୍ଧେଶ: ଅଂଶଗ୍ରହଣକାରୀଙ୍କୁ ପଚାରନ୍ତୁ:  Why are you not taking iron batika (tablets) everyday if pregnant or every week if non pregnant ?  ଯଦି ଆପଣ ଗର୍ଭବତୀ ତେବେ କାହିଁକି ପ୍ରତିଦିନ ଆଇରନ ବଟିକା ଖାଉ ନାହାନ୍ତି, ଯଦି ଆପଣ ଗର୍ଭବତୀ ନୁହନ୍ତି ତେବେ କାହିଁକି ପ୍ରତି ସପ୍ତାହ ଆଇରନ ବଟିକା ଖାଉ ନାହାନ୍ତି?  **DO NOT READ THE ANSWER CHOICES OUT LOUD. ସାମ୍ଭାବ୍ୟ ଉତ୍ତର ଗୁଡିକ ବଡ ପାଟିରେ ପଢନ୍ତୁ ନାହିଁ**  **Check off/click any answer choices stated by the participant.**  ଅଂଶଗ୍ରହଣକାରୀ କହିଥିବା କାରଣ ଗୁଡିକ ଲେଖନ୍ତୁ  **After the participant has finished listing, ask** ଅଂଶଗ୍ରହଣକାରୀ କାରଣ ସବୁ କହି ସାରିଲା ପରେ, ପଚାରନ୍ତୁ: ଆଉ କିଛି?” | 1. Finished the tablets / ବଟିକା ସରିଯାଇଥିଲା.......1  2. Lost the packet / ଔଷଧ ଖୋଳ ହଜି ଯାଇଥିଲା.......2  3. Experienced dark stool / ଗାଢ ରଙ୍ଗର ଝାଡା ହୋଇଥିଲା........3  4. Experienced nausea / ବାନ୍ତି ହେଲାଭଳି ଲାଗିଥିଲା............4  5. Experienced stomach pain / ପେଟରେ ଯନ୍ତ୍ରଣା ଅନୁଭବ କରିଥିଲି....5  6. Fear of big baby / ପେଟରେ ବଢୁଥିବା ଛୁଆର ଆକାର ବଡ ହୋଇଯିବ ବୋଲି ଭୟ ଲାଗିଥିଲା....................6  7. Forgot to take the tablets / ବଟିକା ଗୁଡିକ ଖାଇବାକୁ ଭୁଲିଯାଇଥିଲି.................7  8. Did not see any need/ benefit / ବଟିକା ଖାଇବାର କୌଣସି ଆବଶ୍ୟକତା ବା ଲାଭ ଥିବା ଅନୁଭବ କରି ପାରିନଥିଲି ...........................8  9.Someone advised not to take or to discontinue / ବଟିକା ଖାଇବା ବନ୍ଦ କରିଦେବା ପାଇଁ ବା ନ ଖାଇବା ପାଇଁ କେହି ଜଣେ ଉପଦେଶ ଦେଇଥିଲେ............9  10. Did not like the taste or smell / ବଟିକାର ସ୍ୱାଦ କିମ୍ଭା ଗନ୍ଧ ଭଲ ଲାଗିନଥିଲା.....10  11.Gave tablets to someone else / ବଟିକାଗୁଡିକ ଅନ୍ୟଜଣକୁ ଦେଇ ଦେଇଥିଲି..11  12. End of pregnancy / ଗର୍ଭାବସ୍ଥା ଶେଷ ହେଇଯାଇଥିଲା...........12  13. Wasn’t anemic anymore / ମୋର ରକ୍ତହୀନତା ଆଉନଥିଲା...........13  14. Not in school anymore or school vacation so not taking / ସ୍କୁଲରେ ଆଉ ପଢୁନାହିଁ ବା ସ୍କୁଲ ଛୁଟି ଅଛି, ତେଣୁ ଖାଉ ନାହିଁ  15. I don't need to take tablets everyday / ମୋର ପ୍ରତିଦିନ ଆଇରନ୍ ବଟିକା ଖାଇବା ଦରକାର ନାହିଁ…………………15  16. No one told me to take everyday / ପ୍ରତିଦିନ ଖାଇବା ପାଇଁ ମୋତେ କେହି କହିନାହାନ୍ତି………….16  Did not receive the IFA tablets / ଆଇରନ୍ ବଟିକା ମିଳି ନାହିଁ)……….17  Others / ଅନ୍ୟାନ୍ୟ (ଦର୍ଶାନ୍ତୁ)……….88 |
|  | Have you asked for iron Batika from anyone for your own use in the last 6 months?  **ଗତ 6 ମାସ ମଧ୍ୟରେ ଆପଣ ନିଜର ବ୍ୟବହାର ପାଇଁ କାହାକୁ ଆଇରନ୍ ବଟିକା ମାଗିଛନ୍ତି କି ?** | Yes/ହଁ ………………… 1  No/ନାହିଁ………………....0 |
|  | If Yes, from whom ?  **ଯଦି ହଁ, ତେବେ କାହାକୁ ?** | Anganwadi center/ ଅଙ୍ଗନବାଡି କେନ୍ଦ୍ର……….1  Asha / ଆଶା ………2  Health center / ସ୍ୱାସ୍ଥ୍ୟ କେନ୍ଦ୍ର ………….3  Others / ଅନ୍ୟାନ୍ୟ (ଦର୍ଶାନ୍ତୁ)………..88 |

| Risk Perception ସଙ୍କଟ ସମ୍ୱନ୍ଧୀୟ ଧାରଣା | | |
| --- | --- | --- |
| **Directions: “I am now going to say something. Please tell me if you agree or disagree with it, OK?” Ask participants if they agree with the following statements, or if they are neutral. If they say “agree,” then ask whether they somewhat agree or strongly agree. If they say “disagree”, then ask whether they somewhat disagree or strongly disagree.**  **ନିର୍ଦ୍ଧେଶ: “ବର୍ତ୍ତମାନ ମୁଁ ଆପଣଙ୍କୁ କିଛି ବିବରଣୀ ପଢି କରି ଶୁଣାଇବି \| ଆପଣ ସେଥିରେ ସହମତ କି ଅସହମତ, ଦୟାକରି ମତେ କହିବେ \|” ଅଂଶଗ୍ରହଣକାରୀଙ୍କୁ ପଚାରନ୍ତୁ ଯଦି ସେମାନେ ନିମ୍ନୋକ୍ତ ବିବରଣୀ ଗୁଡିକରେ ସହମତ ନା ନିଷ୍ପେକ୍ଷ ଅଟନ୍ତି \| ଯଦି ସେମାନେ "ସହମତ" ବୋଲି କୁହନ୍ତି, ତାହାଲେ ପଚାରନ୍ତୁ ସେମାନେ କିଛିକାଂଶରେ ସହମତ ନା ଦୃଢଭାବେ ସହମତ \| ଯଦି ସେମାନେ "ଅସହମତ" ବୋଲି କୁହନ୍ତି, ତାହାଲେ ପଚାରନ୍ତୁ ସେମାନେ କିଛିକାଂଶରେ ଅସହମତ ନା ଦୃଢଭାବେ ଅସହମତ”** | | |
|  | Do you think you will become anemic (have lack of blood) in the coming year?  .ଆପଣ ଭାବୁଛନ୍ତି କି, ଆଗାମୀ ଏକବର୍ଷ ମଧ୍ୟରେ ଆପଣଙ୍କୁ ରକ୍ତହୀନତା ହୋଇପାରେ ? | Yes/ହଁ ………………………… 1  No/ନାହିଁ………………....0  I am already anemic/ମୋତେ ପୁର୍ବରୁ ରକ୍ତହୀନତା ହୋଇଅଛି……………………2 |
|  | Do you think someone in your family will become anemic (have lack of blood) in the coming year?  .ଆପଣ ଭାବୁଛନ୍ତି କି, ଆଗାମୀ ଏକବର୍ଷ ମଧ୍ୟରେ ଆପଣଙ୍କ ପରିବାରର କାହାକୁବି ରକ୍ତହୀନତା ହୋଇପାରେ? | Yes/ହଁ ………………………… 1  No/ନାହିଁ………………....0 |
|  | If you became anemic (have lack of blood), it would affect your health in a negative way?  ଯଦି ଆପଣଙ୍କୁ ରକ୍ତହୀନତା ହୁଏ, ଏହା ଆପଣଙ୍କ ସ୍ୱାସ୍ଥ୍ୟ ଉପରେ ଖରାପ ପ୍ରଭାବ ପକାଇବ\| | Strongly disagree / ଦୃଢଭାବେ ଅସହମତ…..1  Disagree / ଅସହମତ…………..2  Neither agree or disagree/ ସହମତନୁହେଁ କି ଅସହମତନୁହେଁ…………………...3  Agree / ସହମତ………………………4  Strongly agree / ଦୃଢଭାବେ ସହମତ…...5 |
|  | If someone in your family became anemic (have lack of blood), it would affect their health in a negative way?  ଯଦି ଆପଣଙ୍କ ପରିବାରରେ କାହାକୁବି ରକ୍ତହୀନତା ହୁଏ, ଏହା ସେମାନଙ୍କ ସ୍ୱାସ୍ଥ୍ୟ ଉପରେ ଖରାପ ପ୍ରଭାବ ପକାଇବ\| | Strongly disagree / ଦୃଢଭାବେ ଅସହମତ…..1  Disagree / ଅସହମତ…………..2  Neither agree or disagree/ ସହମତନୁହେଁ କି ଅସହମତନୁହେଁ…………………...3  Agree / ସହମତ………………………4  Strongly agree / ଦୃଢଭାବେ ସହମତ…...5 |

| Percieved Access ଆଇରନ ବଟିକା ନେଇପାରିବା / ପାଇପାରିବାକୁ ନେଇଥିବା ଧାରଣା | | | |
| --- | --- | --- | --- |
| Show the Iron Batika Tablets**/ଆଇରନ ବଟିକା ଦେଖାଇକରି ପଚାରନ୍ତୁ** | | | |
|  | Do you know where you can get iron batika tablets?  ଆପଣ ଜାଣିଛନ୍ତି କି, କେଉଁଠାରୁ ଆପଣ ଆଇରନ ବଟିକା ପାଇପାରିବେ? | Yes / ହଁ ……………….……………………..1  No / ନାହିଁ …………………......……………0🡺SE1 |  |
|  | Do you agree or disagree with the statement, “It is easy for you (or someone) to get iron batika (tablets)?”  ଏହି ବିବରଣୀ ସହିତ ଆପଣ ସହମତ ନା ଅସହମତ: "ଆପଣ କିମ୍ବା ଅନ୍ୟ କାହାକୁବି ଆଇରନ ବଟିକା ସୁବିଧାରେ ମିଳିପାରେ"  Directions: as above ask about strongly agree or strongly disagree  ନିର୍ଦ୍ଧେଶ: ଆଗରୁ ପଚରା ଯାଇଥିବା ପ୍ରଶ୍ନଗୁଡିକ ପରି, ଏଥିରେ ଦୃଢଭାବେ ସହମତ ନା ଦୃଢଭାବେ ଅସହମତ ପଚାରନ୍ତୁ \| | Strongly disagree / ଦୃଢଭାବେ ଅସହମତ…..1  Disagree / ଅସହମତ…………..2  Neither agree or disagree/ ସହମତନୁହେଁ କି ଅସହମତନୁହେଁ…………………...3  Agree / ସହମତ………………………4  Strongly agree / ଦୃଢଭାବେ ସହମତ…...5 |  |

| Self-Efficacyସ୍ଵ -ସାମର୍ଥ୍ୟ | | |
| --- | --- | --- |
| **Directions: Ask participants if they agree with the following statements, or if they are neutral. If they say “agree,” then ask whether they somewhat agree or strongly agree. If they say “disagree”, then ask whether they somewhat disagree or strongly disagree.**  ନିର୍ଦ୍ଧେଶ: ଅଂଶଗ୍ରହଣକାରୀଙ୍କୁ ପଚାରନ୍ତୁ ଯଦି ସେମାନେ ନିମ୍ନୋକ୍ତ ବିବରଣୀ ଗୁଡିକରେ ସହମତ ନା ନିଷ୍ପେକ୍ଷ ଅଟନ୍ତି \| ଯଦି ସେମାନେ "ସହମତ" ବୋଲି କୁହନ୍ତି, ତାହାଲେ ପଚାରନ୍ତୁ ସେମାନେ କିଛିକାଂଶରେ ସହମତ ନା ଦୃଢଭାବେ ସହମତ \| ଯଦି ସେମାନେ "ଅସହମତ" ବୋଲି କୁହନ୍ତି, ତାହାଲେ ପଚାରନ୍ତୁ ସେମାନେ କିଛିକାଂଶରେ ଅସହମତ ନା ଦୃଢଭାବେ ଅସହମତ” | | |
|  | You can take iron batika tablets every week when you are not pregnant.  ଆପଣ ଗର୍ଭବତୀ ନଥିବା ସମୟରେ ସପ୍ତାହ କୁ ଥରେ ଆଇରନ ବଟିକା ଖାଇପାରିବେ \| | Strongly disagree / ଦୃଢଭାବେ ଅସହମତ…..1  Disagree / ଅସହମତ…………..2  Neither agree or disagree/ ସହମତନୁହେଁ କି ଅସହମତନୁହେଁ…………………...3  Agree / ସହମତ………………………4  Strongly agree / ଦୃଢଭାବେ ସହମତ…...5 |
|  | You believe that you could easily take iron batika  ଆପଣ ବିଶ୍ୱାସ ରଖନ୍ତି ଯେ, ଆପଣ ସହଜରେ/ବିନା ଅସୁବିଧାରେ ଆଇରନ ବଟିକା ଖାଇପାରିବେ \| | Strongly disagree / ଦୃଢଭାବେ ଅସହମତ…..1  Disagree / ଅସହମତ…………..2  Neither agree or disagree/ ସହମତନୁହେଁ କି ଅସହମତନୁହେଁ…………………...3  Agree / ସହମତ………………………4  Strongly agree / ଦୃଢଭାବେ ସହମତ…...5 |
|  | You can take iron batika (tablets) even if your husband/father does not want you to do so  ଆପଣଙ୍କର ସ୍ୱାମୀ / ପିତା ମନାକଲେ ମଧ୍ୟ ଆପଣ ଆଇରନ ବଟିକା ଖାଇପାରିବେ \| | Strongly disagree / ଦୃଢଭାବେ ଅସହମତ…..1  Disagree / ଅସହମତ…………..2  Neither agree or disagree/ ସହମତନୁହେଁ କି ଅସହମତନୁହେଁ…………………...3  Agree / ସହମତ………………………4  Strongly agree / ଦୃଢଭାବେ ସହମତ…...5  Not Applicable / ପ୍ରଯୁଜ୍ୟ ନୁହେଁ……………6 |
|  | You can take iron batika (tablets) even if your mother/mother-in-law does not want you to do so  ଆପଣଙ୍କର ମା / ଶାଶୂ ମନାକଲେ ମଧ୍ୟ ଆପଣ ଆଇରନ ବଟିକା ଖାଇପାରିବେ \| | Strongly disagree / ଦୃଢଭାବେ ଅସହମତ…..1  Disagree / ଅସହମତ…………..2  Neither agree or disagree/ ସହମତନୁହେଁ କି ଅସହମତନୁହେଁ…………………...3  Agree / ସହମତ………………………4  Strongly agree / ଦୃଢଭାବେ ସହମତ…...5  Not Applicable / ପ୍ରଯୁଜ୍ୟ ନୁହେଁ……………6 |

| Outcome Expectations ପରିଣାମର ଆଶା | | |
| --- | --- | --- |
| **Directions: Ask participants if they agree with the following statements, or if they are neutral. If they say “agree,” then ask whether they somewhat agree or strongly agree. If they say “disagree”, then ask whether they somewhat disagree or strongly disagree.**  ନିର୍ଦ୍ଧେଶ: ଅଂଶଗ୍ରହଣକାରୀଙ୍କୁ ପଚାରନ୍ତୁ ଯଦି ସେମାନେ ନିମ୍ନୋକ୍ତ ବିବରଣୀ ଗୁଡିକରେ ସହମତ ନା ନିଷ୍ପେକ୍ଷ ଅଟନ୍ତି \| ଯଦି ସେମାନେ "ସହମତ" ବୋଲି କୁହନ୍ତି, ତାହାଲେ ପଚାରନ୍ତୁ ସେମାନେ କିଛିକାଂଶରେ ସହମତ ନା ଦୃଢଭାବେ ସହମତ \| ଯଦି ସେମାନେ "ଅସହମତ" ବୋଲି କୁହନ୍ତି, ତାହାଲେ ପଚାରନ୍ତୁ ସେମାନେ କିଛିକାଂଶରେ ଅସହମତ ନା ଦୃଢଭାବେ ଅସହମତ” \| | | |
|  | It is important for non pregnant women to take their iron batika (tablets) in one week.  ଗର୍ଭବତୀ ନଥିବା ମହିଳାମାନଙ୍କ ପାଇଁ ଆଇରନ ବଟିକା ପ୍ରତି ସପ୍ତାହରେ ଖାଇବା ଦରକାର \| | Strongly disagree / ଦୃଢଭାବେ ଅସହମତ…..1  Disagree / ଅସହମତ…………..2  Neither agree or disagree/ ସହମତନୁହେଁ କି ଅସହମତନୁହେଁ…………………...3  Agree / ସହମତ………………………4  Strongly agree / ଦୃଢଭାବେ ସହମତ…...5 |
|  | Taking iron batika (tablets) regularly will make you feel stronger.  ନିୟମିତ ଆଇରନ ବଟିକା ଖାଇବା ଦ୍ୱାରା ଆପଣ ଅଧିକ ବଳଥିବା ଅନୁଭବ କରିବେ \| | Strongly disagree / ଦୃଢଭାବେ ଅସହମତ…..1  Disagree / ଅସହମତ…………..2  Neither agree or disagree/ ସହମତନୁହେଁ କି ଅସହମତନୁହେଁ…………………...3  Agree / ସହମତ………………………4  Strongly agree / ଦୃଢଭାବେ ସହମତ…...5 |
|  | Taking iron batika (tablets) every-day while pregnant will not make the baby big.  ଗର୍ଭବତୀ ସମୟରେ ପ୍ରତିଦିନ ଆଇରନ ବଟିକା ଖାଇବା ଦ୍ୱାରା ପେଟରେ ଥିବା ଛୁଆ ବଡ ହୋଇଯିବ ନାହିଁ I | Strongly disagree / ଦୃଢଭାବେ ଅସହମତ…..1  Disagree / ଅସହମତ…………..2  Neither agree or disagree/ ସହମତନୁହେଁ କି ଅସହମତନୁହେଁ…………………...3  Agree / ସହମତ………………………4  Strongly agree / ଦୃଢଭାବେ ସହମତ…...5 |
|  | Taking iron batika (tablets) every-day can help prevent fatigue and dizziness during pregnancy.  ଗର୍ଭାବସ୍ଥା ସମୟରେ ପ୍ରତିଦିନ ଆଇରନ ବଟିକା ଖାଇବା ଦ୍ୱାରା ହାଲିଆ ଏବଂ ମୁଣ୍ଡ ବୁଲାଇବାରୁ ରକ୍ଷା ପାଇପାରିବେ I | Strongly disagree / ଦୃଢଭାବେ ଅସହମତ…..1  Disagree / ଅସହମତ…………..2  Neither agree or disagree/ ସହମତନୁହେଁ କି ଅସହମତନୁହେଁ…………………...3  Agree / ସହମତ………………………4  Strongly agree / ଦୃଢଭାବେ ସହମତ…...5 |

| Intentions ଅଭିଳାଷ | | | |
| --- | --- | --- | --- |
| **Directions: Ask participants if they agree with the following statements, or if they are neutral. If they say “agree,” then ask whether they somewhat agree or strongly agree. If they say “disagree”, then ask whether they somewhat disagree or strongly disagree.**  ନିର୍ଦ୍ଧେଶ: ଅଂଶଗ୍ରହଣକାରୀଙ୍କୁ ପଚାରନ୍ତୁ ଯଦି ସେମାନେ ନିମ୍ନୋକ୍ତ ବିବରଣୀ ଗୁଡିକରେ ସହମତ ନା ନିଷ୍ପେକ୍ଷ ଅଟନ୍ତି \| ଯଦି ସେମାନେ "ସହମତ" ବୋଲି କୁହନ୍ତି, ତାହାଲେ ପଚାରନ୍ତୁ ସେମାନେ କିଛିକାଂଶରେ ସହମତ ନା ଦୃଢଭାବେ ସହମତ \| ଯଦି ସେମାନେ "ଅସହମତ" ବୋଲି କୁହନ୍ତି, ତାହାଲେ ପଚାରନ୍ତୁ ସେମାନେ କିଛିକାଂଶରେ ଅସହମତ ନା ଦୃଢଭାବେ ଅସହମତ” | | | |
|  | If you were to get pregnant in the future, you will take iron batika tablets every-day.  ଯଦି ଆପଣ ଆଗକୁ ଗର୍ଭବତୀ ହେଲେ, ତାହେଲେ ଆପଣ ପ୍ରତିଦିନ ଆଇରନ ବଟିକା ଖାଇବେ \| | Strongly disagree / ଦୃଢଭାବେ ଅସହମତ…..1  Disagree / ଅସହମତ…………..2  Neither agree or disagree/ ସହମତନୁହେଁ କି ଅସହମତନୁହେଁ…………………...3  Agree / ସହମତ………………………4  Strongly agree / ଦୃଢଭାବେ ସହମତ…...5 |  |
|  | You will take iron batika tablets once a week in the future, even if you are not pregnant.  ଆପଣ ଆଗକୁ ଗର୍ଭବତୀ ନଥିଲେ ମଧ୍ୟ, ଆପଣ ସପ୍ତାହକୁ ଥରେ ଆଇରନ ବଟିକା ଖାଇବେ \| | Strongly disagree / ଦୃଢଭାବେ ଅସହମତ…..1  Disagree / ଅସହମତ…………..2  Neither agree or disagree/ ସହମତନୁହେଁ କି ଅସହମତନୁହେଁ…………………...3  Agree / ସହମତ………………………4  Strongly agree / ଦୃଢଭାବେ ସହମତ…...5 |  |
|  | If you are not pregnant you will take iron batika tablets every week even if your husband/male member in your community does not think it is a good idea.ଆପଣଙ୍କ ସ୍ୱାମୀ/ ଆପଣଙ୍କ ସାହି ବା ଗ୍ରାମରେ ଥିବା ପୁରୁଷ ଲୋକଙ୍କୁ ଠିକ୍ ନଲାଗିଲେ ମଧ୍ୟ, ଆପଣ ଗର୍ଭବତୀ ନଥିବା ସମୟରେ ସପ୍ତାହକୁ ଥରେ ଆଇରନ ବଟିକା ଖାଇବେ \| | Strongly disagree / ଦୃଢଭାବେ ଅସହମତ…..1  Disagree / ଅସହମତ…………..2  Neither agree or disagree/ ସହମତନୁହେଁ କି ଅସହମତନୁହେଁ…………………...3  Agree / ସହମତ………………………4  Strongly agree / ଦୃଢଭାବେ ସହମତ…...5 |  |
|  | If you are not pregnant you will take iron batika tablets every week even if your mother-in-law/woman in your community does not think it is a good idea.  ଆପଣଙ୍କ ଶାଶୁ/ଆପଣଙ୍କ ସାହି ବା ଗ୍ରାମରେ ଥିବା ମହିଳାଙ୍କୁ ଠିକ୍ ନଲାଗିଲେ ମଧ୍ୟ, ଆପଣ ଗର୍ଭବତୀ ନଥିବା ସମୟରେ ସପ୍ତାହକୁ ଥରେ ଆଇରନ ବଟିକା ଖାଇବେ \| | Strongly disagree / ଦୃଢଭାବେ ଅସହମତ…..1  Disagree / ଅସହମତ…………..2  Neither agree or disagree/ ସହମତନୁହେଁ କି ଅସହମତନୁହେଁ…………………...3  Agree / ସହମତ………………………4  Strongly agree / ଦୃଢଭାବେ ସହମତ…...5 |  |

| Social Normsସାମାଜିକ ମାନଦଣ୍ଡ | | |
| --- | --- | --- |
| **I will now ask you your thoughts about what you *think* others in this community (hamlet or village) do. This is not what you think they should do but what you think they actually do. Remember that everything is confidential – we will not share any of your answers and we will not ask you for specific names of people in the community. Also, we are just asking for your best guess to each question.**  ଏହି ସମୁଦାୟରେ (ସାହି ବା ଗ୍ରାମରେ), ଆପଣ କଣ ଭବୁଛନ୍ତି ଅନ୍ୟ ଲୋକମାନେ କରନ୍ତି ବୋଲି ମୁଁ ଏବେ ଆପଣଙ୍କୁ ତାହା ବିଷୟରେ ପଚାରିବି \| ଏହା ନୁହେଁ ସେମାନେ କଣ କରିବା କଥା ବୋଲି ଆପଣ ଭାବୁଛନ୍ତି, ମାତ୍ର ଏହା ହେଉଛି ସେମାନେ ପ୍ରକୃତରେ କଣ କରୁଛନ୍ତି ବୋଲି ଆପଣ ଭାବୁଛନ୍ତି \| ମନେରଖନ୍ତୁ ଯେ, ଆପଣ ଦେଇଥିବା ଉତ୍ତରକୁ ଗୋପନୀୟ ରଖାଯିବ ଏବଂ ଆମେ ଏହାକୁ ଅନ୍ୟକାହାକୁ ଜଣାଇବୁ ନାହିଁ \| ଆମେ ଆପଣଙ୍କୁ ଆପଣଙ୍କ ଗ୍ରାମରେ ବା ସାହିରେ ରହୁଥିବା କୌଣସି ନିର୍ଦିଷ୍ଟ ବ୍ୟକ୍ତିଙ୍କର ନାମ କହିବାକୁ କହିବୁ ନାହିଁ \| ପ୍ରତେକ ପ୍ରଶ୍ନ ପାଇଁ ଆମେ ଆପଣଙ୍କୁ ଆପଣଙ୍କ ସର୍ବଶ୍ରେଷ୍ଠ ଅନୁମାନ ଲଗାଇ ଉତ୍ତର ଦେବା ପାଇଁ କହିବୁ \| | | |
|  | What proportion of pregnant women in your community (hamlet or village) take iron batika tablets regularly?  **ଆପଣଙ୍କ ସାହି ବା ଗ୍ରାମରେ କେତେଭାଗ ଗର୍ଭବତୀ ମହିଳା ନିୟମିତ ଭାବେ ଆଇରନ ବଟିକା ଖାଆନ୍ତି \|**  **[READ RESPONSE CHOICES ONLY IF NECESSARY]**\|  **ଯଦି ଆବଶ୍ୟକ ପଡେ, ଦିଆଯାଇଥିବା ଉତ୍ତର ଗୁଡିକ ପଢିକରି ଶୁଣାନ୍ତୁ \|** | None / କେହି ନୁହେଁ ...0  Some / କିଛିଭାଗ…………………..1  About half / ପାଖାପାଖି ଅଧାଭାଗ………………….…....2  Most / **ଅଧିକାଂଶ**ଜଣ………………….…....3  All / ସମସ୍ତେ ………………………4 |
|  | What proportion of Adolescent girl in your community (hamlet or village) take iron batika tablets regularly?  ଆପଣଙ୍କ ସାହି ବା ଗ୍ରାମରେ କେତେଭାଗ କିଶୋରୀ ବାଳିକା ନିୟମିତ ଭାବେ ଆଇରନ ବଟିକା ଖାଆନ୍ତି \|  **[READ RESPONSE CHOICES ONLY IF NECESSARY]**\|  **ଯଦି ଆବଶ୍ୟକ ପଡେ, ଦିଆଯାଇଥିବା ଉତ୍ତର ଗୁଡିକ ପଢିକରି ଶୁଣାନ୍ତୁ \|** | None / କେହି ନୁହେଁ ...0  Some / କିଛିଭାଗ…………………..1  About half / ପାଖାପାଖି ଅଧାଭାଗ………………….…....2  Most / **ଅଧିକାଂଶ**ଜଣ………………….…....3  All / ସମସ୍ତେ ………………………4 |
| I will now ask you your thoughts about what you think others in this community (hamlet or village)*should* do. This is not what you think they actually do, this time I am asking only about what you think they should do.ମୁଁଏବେଆପଣଙ୍କୁ, ଏହିସମୁଦାୟର(ସାହି ବା ଗ୍ରାମର)ଅନ୍ୟଲୋକମାନେକଣକରିବାଉଚିତବୋଲିଭାବୁଛନ୍ତିସେ ବିଷୟରେପଚାରିବି \| ସେମାନେପ୍ରକୃତରେକଣକରୁଛନ୍ତିବୋଲିଆପଣଭାବୁଛନ୍ତିତାହା ବିଷୟରେ ମୁଁ ପଚାରିବି ନାହିଁ \|ମାତ୍ରସେମାନେକଣକରିବାଉଚିତବୋଲିଆପଣଭାବୁଛନ୍ତିତାହା ବିଷୟରେ ମୁଁ ପଚାରିବି \| | | |
|  | What proportion of non pregnant women in your community (hamlet or village) take iron batika tablets regularly.  ଆପଣଙ୍କ ସାହି ବା ଗ୍ରାମରେ କେତେଭାଗ ଗର୍ଭବତୀ ନଥିବା ମହିଳା ନିୟମିତଭାବେ ଆଇରନ ବଟିକା ଖାଆନ୍ତି | None / କେହି ନୁହେଁ ...0  Some / କିଛିଭାଗ…………………..1  About half / ପାଖାପାଖି ଅଧାଭାଗ………………….…....2  Most / **ଅଧିକାଂଶ**ଜଣ………………….…....3  All / ସମସ୍ତେ ………………………4 |
|  | How many women in your community (hamlet or village) think you should take iron batika tablets regularly if you are pregnant.  ଆପଣଙ୍କ ସାହି ବା ଗ୍ରାମରେ, କେତେଭାଗ ମହିଳା ଭାବନ୍ତି ଯେ, ଆପଣ ଗର୍ଭବତୀ ଥିବା ସମୟରେ ନିୟମିତଭାବେ ଆଇରନ ବଟିକା ଖାଇବା ଉଚିତ \| | None / କେହି ନୁହେଁ ...0  Some / କିଛିଭାଗ…………………..1  About half / ପାଖାପାଖି ଅଧାଭାଗ………………….…....2  Most / **ଅଧିକାଂଶ**ଜଣ………………….…....3  All / ସମସ୍ତେ ………………………4 |
|  | How many of the women in your community (hamlet or village) think you should take IRON BATIKA tablets, even when you are not pregnant.  ଆପଣଙ୍କ ସାହି ବା ଗ୍ରାମରେ, କେତେଭାଗ ମହିଳା ଭାବନ୍ତି ଯେ, ଆପଣ ଗର୍ଭବତୀ ନଥିବା ସମୟରେ ମଧ୍ୟ ନିୟମିତଭାବେ ଆଇରନ ବଟିକା ଖାଇବା ଉଚିତ \|  If she is not married, say her mother  ଯଦିଉତ୍ତରଦାତାବିବାହିତାନୁହଁନ୍ତି, ତାହାଲେଶାଶୁବଦଳରେମାବ୍ୟବହାରକରିଏହିପ୍ରଶ୍ନଟିପଚାରନ୍ତୁ \| | None / କେହି ନୁହେଁ ...0  Some / କିଛିଭାଗ…………………..1  About half / ପାଖାପାଖି ଅଧାଭାଗ………………….…....2  Most / **ଅଧିକାଂଶ**ଜଣ………………….…....3  All / ସମସ୍ତେ ………………………4 |
|  | For married women :  Your mother-in-law thinks you should take iron batika tablets regularly if you are pregnant.  ବିବାହିତା ମହିଳା ମାନଙ୍କ ପାଇଁ :  ଆପଣ ଗର୍ଭବତୀ ଥିବା ସମୟରେ, ନିୟମିତଭାବେ ଆଇରନ ବଟିକା ଖାଇବା ଉଚିତ ବୋଲି ଆପଣଙ୍କ ଶାଶୂ ଭାବନ୍ତି \|  For unmarried women:  Most mother-in-law think pregnant women should take iron batika tablets regularly. ଅବିବାହିତା ମହିଳା ମନଙ୍କ ପାଇଁ :  ଅଧିକାଂଶ ଶାଶୁମାନେ ଭାବନ୍ତି ଯେ ଗର୍ଭବତୀ ମହିଳାମାନେ ନିୟମିତ ଭାବେ ଆଇରନ ବଟିକା ଖାଇବା ଉଚିତ \|  If she is not married, say her mother  ଯଦିଉତ୍ତରଦାତାବିବାହିତାନୁହଁନ୍ତି, ତାହାଲେଶାଶୁବଦଳରେମାବ୍ୟବହାରକରିଏହିପ୍ରଶ୍ନଟିପଚାରନ୍ତୁ \| | Strongly disagree / ଦୃଢଭାବେ ଅସହମତ…..1  Disagree / ଅସହମତ…………..2  Neither agree or disagree/ ସହମତନୁହେଁ କି ଅସହମତନୁହେଁ…………………...3  Agree / ସହମତ………………………4  Strongly agree / ଦୃଢଭାବେ ସହମତ…...5 |
|  | For married women :  Your mother-in-law thinks you should take iron batika tablets regularly, even if you are not pregnant.  ବିବାହିତା ମହିଳା ମାନଙ୍କ ପାଇଁ :  ଆପଣ ଗର୍ଭବତୀ ନଥିବା ସମୟରେ ମଧ୍ୟ, ନିୟମିତଭାବେ ଆଇରନ ବଟିକା ଖାଇବା ଉଚିତ ବୋଲି ଆପଣଙ୍କ ଶାଶୂ ଭାବନ୍ତି  For unmarried women :  Most mother-in-law think non pregnant women should take iron batika tablets.  ଅବିବାହିତା ମହିଳା ମନଙ୍କ ପାଇଁ :  ଅଧିକାଂଶ ଶାଶୁମାନେ ଭାବନ୍ତି ଯେ ମହିଳାମାନେ ଗର୍ଭବତୀ ନଥିବା ସମୟରେ ମଧ୍ୟ ନିୟମିତ ଭାବେ ଆଇରନ ବଟିକା ଖାଇବା ଉଚିତ \|  If she is not married, say her mother  ଯଦିଉତ୍ତରଦାତାବିବାହିତାନୁହଁନ୍ତି, ତାହାଲେଶାଶୁବଦଳରେମାବ୍ୟବହାରକରିଏହିପ୍ରଶ୍ନଟିପଚାରନ୍ତୁ \| | Strongly disagree / ଦୃଢଭାବେ ଅସହମତ…..1  Disagree / ଅସହମତ…………..2  Neither agree or disagree/ ସହମତନୁହେଁ କି ଅସହମତନୁହେଁ…………………...3  Agree / ସହମତ………………………4  Strongly agree / ଦୃଢଭାବେ ସହମତ…...5 |
|  | For married women :  Your husband thinks you should take iron batika tablets regularly if you are pregnant  ବିବାହିତା ମହିଳା ମାନଙ୍କ ପାଇଁ :  ଆପଣ ଗର୍ଭବତୀ ଥିବା ସମୟରେ, ନିୟମିତଭାବେ ଆଇରନ ବଟିକା ଖାଇବା ଉଚିତ ବୋଲି ଆପଣଙ୍କ ସ୍ୱାମୀ ଭାବନ୍ତି \|  For unmarried women:  Most husband think pregnant women should take iron batika tablets regularly.  ଅବିବାହିତା ମହିଳା ମନଙ୍କ ପାଇଁ :  ଅଧିକାଂଶ ସ୍ୱାମୀମାନେ ଭାବନ୍ତି ଯେ ଗର୍ଭବତୀ ମହିଳାମାନେ ନିୟମିତ ଭାବେ ଆଇରନ ବଟିକା ଖାଇବା ଉଚିତ \|  If she is not married, say her mother  ଯଦିଉତ୍ତରଦାତାବିବାହିତାନୁହଁନ୍ତି, ତାହାଲେଶାଶୁବଦଳରେମାବ୍ୟବହାରକରିଏହିପ୍ରଶ୍ନଟିପଚାରନ୍ତୁ \| | Strongly disagree / ଦୃଢଭାବେ ଅସହମତ…..1  Disagree / ଅସହମତ…………..2  Neither agree or disagree/ ସହମତନୁହେଁ କି ଅସହମତନୁହେଁ…………………...3  Agree / ସହମତ………………………4  Strongly agree / ଦୃଢଭାବେ ସହମତ…...5 |
|  | For married women Your husband thinks you should take iron batika tablets regularly when you are not pregnant  ବିବାହିତା ମହିଳା ମାନଙ୍କ ପାଇଁ:  ଆପଣ ଗର୍ଭବତୀ ନଥିବା ସମୟରେ ମଧ୍ୟ, ନିୟମିତଭାବେ ଆଇରନ ବଟିକା ଖାଇବା ଉଚିତ ବୋଲି ଆପଣଙ୍କ ସ୍ୱାମୀଭାବନ୍ତି\|  For unmarried women Most husband think non pregnant women should take iron batika tablets regularly.  ଅବିବାହିତା ମହିଳା ମନଙ୍କ ପାଇଁ :  ଅଧିକାଂଶ ସ୍ୱାମୀମାନେ ଭାବନ୍ତି ଯେ ମହିଳାମାନେ ଗର୍ଭବତୀ ନଥିବା ସମୟରେ ମଧ୍ୟ ନିୟମିତ ଭାବେ ଆଇରନ ବଟିକା ଖାଇବା ଉଚିତ \|  ଉତ୍ତରଦାତାବିବାହିତାନୁହଁନ୍ତି, ତେବେ ତାଙ୍କ ସ୍ୱାମୀଙ୍କ ବଦଳରେ ବାପା ବ୍ୟବହାର କରି ଏହି ପ୍ରଶ୍ନଟି ପଚାରନ୍ତୁ | Strongly disagree / ଦୃଢଭାବେ ଅସହମତ…..1  Disagree / ଅସହମତ…………..2  Neither agree or disagree/ ସହମତନୁହେଁ କି ଅସହମତନୁହେଁ…………………...3  Agree / ସହମତ………………………4  Strongly agree / ଦୃଢଭାବେ ସହମତ…...5 |

| Knowledge about IRON BATIKA and other conditionsଆଇରନ ବଟିକା ଏବଂ ଅନ୍ୟ ସ୍ଥିତି ଉପରେ ଜ୍ଞାନ | | |
| --- | --- | --- |
| **Directions: Say** “I’m going to read you a number of statements. After I finish reading each statement, please tell me if you think it is true or not.”  **ନିର୍ଦ୍ଧେଶ:** କୁହନ୍ତୁ "ମୁଁ ଆପଣଙ୍କୁ କେତେଗୁଡିଏ ବିବରଣୀ ପଢିକରି ଶୁଣାଇବାକୁ ଯାଉଛି \| ମୁଁ ପତ୍ୟେକଟି ବିବରଣୀ ପଢି ସାରିଲା ପରେ, ଦୟାକରି ମତେ କୁହନ୍ତୁ ଆପଣ ଏହା ସତ କି ନାହିଁ ବୋଲି ଭାବୁଛନ୍ତି \|" | | |
|  | Iron batika prevents anemia (lack of blood) only for young women and it has no effect on older women.  ଆଇରନ ବଟିକା କେବଳ କମ୍ ବୟସର ମହିଳା ମାନଙ୍କୁ ରକ୍ତହୀନତା ହେବାରୁ ରକ୍ଷାକରେ ଏବଂ ବୟସ୍କ ମହିଳା ମାନକ ଉପରେ ଆଇରନ ବଟିକାର ପ୍ରଭାବ ନଥାଏ \| | True ସତ୍ୟ ………………………….1  False ମିଥ୍ୟା ………………………..2  Don’t know ଜଣା ନାହିଁ …………..3 |
|  | Anemia (lack of blood) can be cured by exercising more..  ଅଧିକ ବ୍ୟାୟାମ କରିବା ଦ୍ବାରା ରକ୍ତହୀନତାକୁ ଦୁର କରି ହେବ \| | True ସତ୍ୟ ………………………….1  False ମିଥ୍ୟା ………………………..2  Don’t know ଜଣା ନାହିଁ …………..3 |
|  | Eating dark green leafy vegetables prevents anemia, or lack of blood.  ଗାଢ଼ ସବୁଜ ରଙ୍ଗର ପତ୍ର ଥିବା ପନିପରିବା ଖାଇଲେ ଏହା ରକ୍ତହୀନତା ବା ଆନେମିଆ ହେବାରୁ ରକ୍ଷାକରେ \| | True ସତ୍ୟ ………………………….1  False ମିଥ୍ୟା ………………………..2  Don’t know ଜଣା ନାହିଁ …………..3 |
|  | Having malaria can make it easier to get anemia or lack of blood.  ମ୍ୟାଲେରିଆ ଥିଲେ, ରକ୍ତହୀନତା ବା ଆନେମିଆ ସହଜରେ ହୋଇଥାଏ \| | True ସତ୍ୟ ………………………….1  False ମିଥ୍ୟା ………………………..2  Don’t know ଜଣା ନାହିଁ …………..3 |
|  | Routine deworming can reduce anemia or lack of blood  ନିୟମିତ କୁର୍ମୀନାଶକ ଖାଇଲେ ରକ୍ତହୀନତା ବା ଆନେମିଆ କମ ହୋଇପାରିବ \| | True ସତ୍ୟ ………………………….1  False ମିଥ୍ୟା ………………………..2  Don’t know ଜଣା ନାହିଁ …………..3 |
|  | Anemia can be spread from one person to another through their saliva  ଆନେମିଆ ଜଣେ ଲୋକଠାରୁ ଅନ୍ୟ ଲୋକପାଖକୁ ସେମାନଙ୍କ ଲାଳ ମାଧ୍ୟମରେ ବ୍ୟାପିଥାଏ \| | True ସତ୍ୟ ………………………….1  False ମିଥ୍ୟା ………………………..2  Don’t know ଜଣା ନାହିଁ …………..3 |
|  | Eating rice and chapati can prevent anemia.  ଭାତ ଏବଂ ରୁଟି ଖାଇଲେ ରକ୍ତହୀନତା ବା ଆନେମିଆ ହେବାରୁ ରକ୍ଷା କରିପାରିବ \| | True ସତ୍ୟ ………………………….1  False ମିଥ୍ୟା ………………………..2  Don’t know ଜଣା ନାହିଁ …………..3 |

| Demographics ଜନପଦ ସମ୍ୱନ୍ଧୀୟତଥ୍ୟ | | |
| --- | --- | --- |
| Thank you so much for answering my questions about anemia and IRON BATIKA. I’d like to ask you a few questions about yourself before we finish.  ଆନେମିଆ ଏବଂ ଆଇରନ ବଟିକା ବିଷୟରେ ମୋର ପ୍ରଶ୍ନର ଉତ୍ତର ଦେଇଥିବାରୁ ଆପଣଙ୍କୁ ବହୁତ ଧନ୍ୟବାଦ \| ଏହି ସାକ୍ଷାତକାରଟିକୁ ଶେଷ କରିବା ପୂର୍ବରୁ ମୁଁ ଆପଣଙ୍କୁ ଆପଣଙ୍କ ନିଜ ବିଷୟରେ କିଛି ପ୍ରଶ୍ନ ପଚାରିବା ପାଇଁ ଚାହୁଁଛି \| | | |
|  | What is your age in years?  ଆପଣଙ୍କବୟସକେତେ (ବର୍ଷରେ)  Unknown/ଜଣାନାହିଁ: 999 | Age/ବୟସ: ________________ (years)  Unknown/ଜଣାନାହିଁ: 999 |
|  | What is the highest level of school you have attended?  ଆପଣ ବିଦ୍ୟାଳୟରେ ପଢିଥିବା ସର୍ବୋଚ୍ଚ ଶ୍ରେଣୀ କେତେ ? | None/ପଢିନାହାନ୍ତି ------- 0  Class-1 (completed) /ପ୍ରାଥମ ଶ୍ରେଣୀ (ସମ୍ପୁର୍ଣ) -----1  Class-2 (completed) /ପ୍ରାଥମ ଶ୍ରେଣୀ (ସମ୍ପୁର୍ଣ)----- 2  Class-3 (completed) /ପ୍ରାଥମ ଶ୍ରେଣୀ (ସମ୍ପୁର୍ଣ)----- 3  Class-4 (completed) /ପ୍ରାଥମ ଶ୍ରେଣୀ (ସମ୍ପୁର୍ଣ)----- 4  Class-5 (completed) /ପ୍ରାଥମ ଶ୍ରେଣୀ (ସମ୍ପୁର୍ଣ) ----- 5  Class-6 (completed) /ପ୍ରାଥମ ଶ୍ରେଣୀ (ସମ୍ପୁର୍ଣ)----- 6  Class-7 (completed) /ପ୍ରାଥମ ଶ୍ରେଣୀ (ସମ୍ପୁର୍ଣ) ----- 7  Class-8 (completed) /ପ୍ରାଥମ ଶ୍ରେଣୀ (ସମ୍ପୁର୍ଣ)-- 8  Class-9 (completed) /ପ୍ରାଥମ ଶ୍ରେଣୀ (ସମ୍ପୁର୍ଣ) ----- 9  Class-10 (completed) /ପ୍ରାଥମ ଶ୍ରେଣୀ (ସମ୍ପୁର୍ଣ)--- 10  Class-11 (completed) /ପ୍ରାଥମ ଶ୍ରେଣୀ (ସମ୍ପୁର୍ଣ)--- 11  Class-12 (completed) /ପ୍ରାଥମ ଶ୍ରେଣୀ (ସମ୍ପୁର୍ଣ)--- 12  More than Class-12 (completed)/ଦ୍ୱାଦଶରୁ ଅଧିକ ଶ୍ରେଣୀ (ସମ୍ପୁର୍ଣ)--- 13  No response/ଉତ୍ତର ଦେଲେ ନାହିଁ ----------- 99 |
|  | What is your religion?  ଆପଣଙ୍କଧର୍ମକଣ ? | Hindu/ହିନ୍ଦୁ -----------------------------1  Muslim/ମୁସଲମାନ୍ ---------------------2  Christian/ଖ୍ରୀଷ୍ଟିଆନ୍ ---------------------3  Sikh/ଶିଖ୍ -------------------------------4  Buddhist/ବୌଦ୍ଧ ------------------------5  Jain/ଜୈନ ------------------------------6  Other/ଅନ୍ୟାନ୍ୟ -------------------------7 |
|  | Are you a part of a caste or tribe?  ଆପଣ କୌଣସି ଜାତି ନା ଜନଜାତିରେ ଅନ୍ତର୍ଭୁକ୍ତ କି ? | Yes /ହଁ………………………1  No /ନା ……………………….0  I don’t know /ମୁଁ ଜାଣି ନାହିଁ ……….99 |
|  | Is this a scheduled caste, a scheduled tribe, other backward class, or none of them?  ଆପଣ ଗୋଟିଏ ଅନୁସୂଚିତ ଜାତି ନା ଅନୁସୂଚିତ ଜନଜାତି ନା ଅନ୍ୟ ପଛୁଆ ବର୍ଗରେ ଆସୁଛନ୍ତି କି ନା ଏଥି ମଧ୍ୟରୁ କେଉଁଟିରେ ଆସୁନାହାଁନ୍ତି ? | Scheduled Caste/ଅନୁସୂଚିତଜାତି ----------1  Scheduled Tribe/ଅନୁସୂଚିତଜନଜାତି ------2  OBC/ଅନ୍ୟାନ୍ୟପଛୁଆବର୍ଗ -----------------3  None of themଏଥି ମଧ୍ୟରୁ କେଉଁଟିବି ନୁହେଁ …....4  Don’t knowଜଣା ନାହିଁ ………………5 |
|  | How many children do you have?  ଆପଣଙ୍କର କେତେ ଗୋଟି ପିଲା ଅଛନ୍ତି ? | None/କେହି ନାହାନ୍ତି ------0  One/ଏକ ----------------1  Two/ଦୁଇ ----------------2  Three/ତିନି --------------3  Four/ଚାରି ---------------4  Five or more/ପାଞ୍ଚ ବା ଅଧିକା ----------5  No response/ ଉତ୍ତରଦେଲେନାହିଁ -----99 |
|  | Do you own a mobile phone?  ଆପଣଙ୍କପାଖରେ ମୋବାଇଲ୍ ଫୋନ୍ ଅଛି କି ? | Yes/ହଁ……………...1🡪Skip to Dem11  No/ନା………...…….0 |
|  | Whose phone do you use most?  ଆପଣ କାହା ଫୋନ୍ ସବୁଠୁ ଅଧିକ ବ୍ୟବହାର କରୁଛନ୍ତି ? | Husband/Male Partner  ସ୍ବାମୀ/ପୁରୁଷ ସାଥୀ---------- 1  Mother/Mother-in-law ମା/ଶାଶୁ-------- 2  Father/Father-in-law ବାପା/ଶଶୁର------ 3  Male relativeପୁରୁଷ ସମ୍ପର୍କୀୟ--------------4  Female relative ମହିଳା ସମ୍ପର୍କୀୟ-----------5  Friend ବନ୍ଧୁ-------------6  Children ପିଲାମାନେ--------------------- 7  I never use any phone ମୁଁ କେବେ ଫୋନ୍ ବ୍ୟବହାର କରିନାହିଁ ……………………..8  Other ଅନ୍ୟାନ୍ୟ ------------- 88 |
|  | How often do you have to ask for permission to use mobile phones?  ମୋବାଇଲ ଫୋନ୍ ବ୍ୟବହାର କରିବାପାଇଁ ଆପଣ କେତେଥର ଅନୁମତି ନିଅନ୍ତି ? | Always/ସବୁବେଳେ ………1  Most of the times/ଅଧିକାଂଶ ସମୟ……2  Rarely/କୋଚିତ୍……………..3  Never/କେବେ ନୁହେଁ………4 |
|  | From whom do you need to ask for permission?  କାହାଠାରୁ ଅନୁମତି ନେବା ନିହାତି ଆବଶ୍ୟକ ଅଟେ ? | Husband/Male Partner  ସ୍ବାମୀ/ପୁରୁଷ ସାଥୀ ………… 1  Mother/Mother-in-law ମା/ଶାଶୁ………………… 2  Father/Father-in-law ବାପା/ଶଶୁର ………………… 3  Male relative/ପୁରୁଷ ସମ୍ପର୍କୀୟ …..………4  Female relative ମହିଳା ସମ୍ପର୍କୀୟ…………………5  Friend ବନ୍ଧୁ…………………6  Children ପିଲାମାନେ…………………7  I never use any phone ମୁଁ କେବେ ଫୋନ୍ ବ୍ୟବହାର କରିନାହିଁ ……………………..8  Other ଅନ୍ୟାନ୍ୟ ………… 88 |
|  | What is the number of mobile (phone) you use?  ଆପଣବ୍ୟବହାର କରୁଥିବା ମୋବାଇଲ୍ ଫୋନ୍ ନମ୍ବରଟି କଣ ?  Instruction: If she has more than one number, ask her to tell the number that is mostly used by her.  ଯଦି ତାଙ୍କର ଏକରୁ ଅଧିକ ମୋବାଇଲ ନମ୍ବର ଥାଏ, ଅଧିକାଂଶ ସମୟରେ ବ୍ୟବହାର କରୁଥିବା ତାଙ୍କ ମୋବାଇଲ ନମ୍ବରଟିକୁ ଲେଖନ୍ତୁ \| |  |
|  | Do you share the phone with anyone?  ଆପଣ କାହାକୁ ଫୋନଟିକୁ ବ୍ୟବହାର କରିବା ପାଇଁ ଦିୟନ୍ତି କି?  (Only ask if Dem7 is ‘Yes’) | Yes/ହଁ ……………………..…1  No/ନା …………………..…...0 |
|  | Who do you share most your phone with?  ଆପଣ ଅଧିକ ଥର କାହାକୁ ଫୋନଟିକୁ ବ୍ୟବହାର କରିବା ପାଇଁ ଦିୟନ୍ତି? | Husband/Male Partner  ସ୍ବାମୀ/ପୁରୁଷ ସାଥୀ--------- 1  Mother/Mother-in-law ମା/ଶାଶୁ-------- 2  Father/Father-in-law ବାପା/ଶଶୁର------- 3  Male relative/ପୁରୁଷ ସମ୍ପର୍କୀୟ----------4  Female relative ମହିଳା ସମ୍ପର୍କୀୟ-----------5  Friend ବନ୍ଧୁ-------------6  Children ପିଲାମାନେ---- 7  I never use any phone ମୁଁ କେବେ ଫୋନ୍ ବ୍ୟବହାର କରିନାହିଁ ……………………..8  Other ଅନ୍ୟାନ୍ୟ -------- 88 |
|  | Which of the following activities are you comfortable doing on your own?  କାର୍ଯ୍ୟବଳି ମଧ୍ୟ୍ୟରୁ କେଉଁ ଗୁଡିକ ନିଜେ କରିବାରେ ଆରାମଦାୟକ ମନେକରନ୍ତି? | Make a call ଫୋନ୍ କରିବା….1  Receive a call କଲ ଗ୍ରହଣ କରିବା….2  Send an SMS ବାର୍ତା ପଠେଇବା…….3  Receive an SMS ବାର୍ତା ଗ୍ରହଣ କରିବା……4  Deliberate missed call ମିସ୍ କଲ କରିବା……5  Use the internet ଇଣ୍ଟେରନେଟର ବ୍ୟବହାର….6  Use WhatsApp ହ୍ୱାଟସାପର ବ୍ୟବହାର……………7  Use Facebook ଫେସବୂକ୍ ର ବ୍ୟବହାର…………………8  Nothing କୌଣସି ନୁହେଁ…………….9  Other ଅନ୍ୟାନ୍ୟ………………….88 |
|  | Can you give us the name and phone number of someone who will always know where you are, in case you move in the next two years?  ଆପଣ ଏମିତି ଜଣଙ୍କର ନାମ ଏବଂ ଫୋନନମ୍ବର ଦିୟନ୍ତୁ ଯିଏ ଆପଣ ଆଗାମୀ ଦୁଇ ବର୍ଷରେ ବାହାରକୁ ଗଲେ ମଧ୍ୟ, ଆପଣ କେଉଁଠାରେ ଅଛନ୍ତି ସେ କହି ପାରିବେ \| | Name of person  ବ୍ୟକ୍ତିଙ୍କର ନାମ:  Mobile number  ମୋବାଇଲ ନମ୍ବର: |
|  | Are you currently a part of a self-help group (SHG)?  ବର୍ତ୍ତମାନ ଆପଣ ଗୋଟିଏ ସ୍ୱଂୟ ସହାୟକ ଗୋଷ୍ଠୀର ଏକ ସଦସ୍ୟ କି ? | Yes/ହଁ ……………………..…1  No/ନା …………………..…...0 |
|  | Has a doctor, nurse, ANM, ASHA, or healthcare provider ever told you that you have anemia?  ଆପଣଙ୍କର ଆନେମିଆ ଅଛି ବୋଲି, ଜଣେ ଡାକ୍ତର କିମ୍ଭା ନର୍ସ କିମ୍ଭା ଆଶା କିମ୍ଭା ସ୍ୱାସ୍ଥ୍ୟ ସେବାଦାତା ଆପଣଙ୍କୁ କେବେବି ଜଣାଇଥିଲେ କି ? | Yes/ହଁ ……………………..…1  No/ନା …………………..…...0 |
|  | Do you currently have anemia or lack of blood?  ଆପଣଙ୍କର ବର୍ତ୍ତମାନ ଆନେମିଆ ବା ରକ୍ତହୀନତା ଅଛି କି ? | Yes/ହଁ ……………………..…1  No/ନା …………………..…...0  don’t know ମୁଁ ଜାଣି ନାହିଁ ………….…..99 |
|  | Have you been treated with deworming medication in the past year?  ଗଲା ବର୍ଷ ଭିତରେ, ଆପଣଙ୍କର କୃମିନାଶକ ଚିକିତ୍ସା କରାଯାଇଛି କି ? | Yes/ହଁ ……………………..…1  No/ନା…………………..…...0  don’t know/ ମୁଁ ଜାଣି ନାହିଁ …..99 |
|  | Have you had malaria in the past 6 months?  ଗତ 6 ମାସ ଭିତରେ, ଆପଣଙ୍କୁ ମ୍ୟାଲେରିଆ ହୋଇଛି କି ? | Yes/ହଁ ……………………..…1  No/ନା …………………..…...0  I don’t know ମୁଁ ଜାଣି ନାହିଁ ………….…..99 |
|  | Have you had diarrhea in the past 4 weeks?  ଗତ 4 ସପ୍ତାହ ଭିତରେ, ଆପଣଙ୍କୁ ତରଳ ଝାଡା ହୋଇଛି କି ? | Yes/ହଁ ……………………..…1  No/ନା …………………..…...0  I don’t know ମୁଁ ଜାଣି ନାହିଁ ………….…..99 |
|  | Do you regularly consume tea at the time of or within an hour of eating a meal (like lunch or dinner)?  ଆପଣ ନିୟମିତ ଭାବେ ଭୋଜନ ଖାଇଲା ବେଳେ ବା ଭୋଜନ ଖାଇବାର ଏକ ଘଣ୍ଟା ଭିତରେ ଚାହା ପିଅନ୍ତି କି (ଯେପରିକି ମଧ୍ୟାହ୍ନ ବା ରାତ୍ରି ଭୋଜନ) ? | Yes/ହଁ ……………………..…1  No/ନା …………………..…...0 |
