# Supplementary material for: Examining intentions to take iron supplements to inform a behavioral intervention: The Reduction in Anemia through Normative Innovations (RANI) project
Source: PLoS One. 2021 May 11;16(5):e0249646. doi: 10.1371/journal.pone.0249646 (PMC8112683; doi:10.1371/journal.pone.0249646)
Supplement: Attachment — Submitted filename: PONE-D-20-27062 review.docx [file pone.0249646.s002.docx]

The paper examines the effect of descriptive, injunctive and collective norms on the intention to take iron tablets among a sample of non-pregnant women in India. Using a well-designed survey, the authors find that, in this community where few women take iron tablets, descriptive norms are not very strong, but injunctive and collective norms have a moderate influence on intention. We already know from other research that there is a strong relationship between intention and behaviour. The authors plan to use this information to design a norms intervention that will be tested in a subsequent RCT.

These findings add useful knowledge to the literature on social norms, by demonstrating the importance of understanding local context before embarking on a social norms intervention. They also provide a nice example of using baseline data for a RCT to inform the design of the intervention that will be tested in the RCT, which is an efficient study design I have not come across before.

The conceptual framework relies heavily on the theory of normative social behaviour and other work written by the authors themselves. The authors have clearly done a substantial amount of work in this area. However, the conceptual framework could be strengthened by reference to other relevant theories, such as social identity theory, theory of planned behaviour, theory or normative conduct, to place the work in a wider context.

Overall the survey and analysis methods are appropriate and well-described. I have a few suggestions to improve the methods reporting:

- Line 100. Change future tense to past tense.
- Line 119-121. The final sentence of the sampling method was not quite clear to me. I think it may describe some stratification by caste and tribe, but this was not clear because the word stratification is not used. Description of the caste and tribe variables, in particular how many categories there are, would help with clarity.
- A power calculation is needed. This study describes the analysis of the baseline data from a RCT. I assume the authors did a sample size calculation for the RCT, which would not be appropriate to report here. However, I would like to see some reassurance that the size of the sample is sufficient for the analysis that was undertaken.
- Line 164-166. The start of this sentence indicates that the questions quoted later in the sentence are all Yes/No questions, but the first one requires a number as the answer – this just needs re-phrasing.
- The collective norms variable was created from responses to the Intention to take iron question. The section on collective norms (lines 173-179) is difficult to understand without the reader having prior knowledge of the survey question about Intentions. This could be resolved by moving the section on Intentions (line 211-221) to become the first variable in the list.
- Line 194-198. Briefly explain the rationale for including a question about pregnancy, when non3 of the women were pregnant.
- Line 218-221. I am not quite clear from this description what transformation was done. Did the authors try out a number of different options and find that squaring it was the most suitable approach? Or did they do something else and then square that? The Stata commands gladder and ladder can be helpful in selecting a suitable transformation. I am not suggesting the authors should use those commands, simply that it be explained more clearly.
- Pearson’s correlations are always zero-ordered, i.e. they assess the level of correlation between two variables without reference to other variables. I don’t think it is necessary to include the term zero-ordered, and that term may confuse people who are not used to seeing it.
- Missing data - What proportion of the women who were identified for the study consented to join it? The amount of missing data appears to be very low. Out of 3914 people in the sample, 3793 were included in the analysis. I think this warrants mention – what was done to achieve such high responses?

A few suggestions for clarity in presenting the results:

- Table 1. It would be informative to include the collective norms measure in the table and the associated text.
- Table 2. The table includes the correlations between all of the variables in the model, but only the correlations with the outcome are reported in the text. I think it would be useful to briefly summarise the associations between the other variables, i.e. reassuringly, there are no high levels of correlation that might be worrying.
- Table 3. Add standard errors and confidence intervals for all variables. Make clear in the table whether the coefficients are standardised beta or not. I saw something in the text which suggested these were standardised, but I am not sure looking at this whether they are. The benefit of using the original scales is that the coefficients can be more easily interpreted, but standardised results offer the opportunity to compare between the variables. If the journal allows, you could add the standardised results as a supplementary file.

Discussion:

Injunctive norms are operationalised in the survey as ‘influential referent groups’ in the women’s local communities (women, husbands, mothers-in-law, mothers). In the discussion (line 326-339) the authors suggest that informing women and their families that the Indian government recommends iron would lead to women believing they should take it. It seems rather a leap from finding that women are influenced by the expectations of their local community and family to suggesting that they will be influence by the expectations of the government. These are injunctive norms operating at two different levels and I don’t think the second level was asked about in the survey. It may be that the authors think that a government recommendation would influence the husbands, mother-in-law and mothers, or that the recommendation will work in some other way, but I didn’t find that clear in the paper.

Conclusion:

If I am reading Table 3 correctly (if the results are standardised) the variable with the largest effect was self-efficacy. This is mentioned in the text, but I think it would also be useful to mention this in the Conclusion and also in the Abstract. It seems to suggest another possible intervention that would be worth pursuing in this context.

There is no mention of a published protocol, but it is not usual to publish a protocol for a survey.

This analysis used data that forms the baseline of a RCT. The authors plan to deposit the data in a repository, one the trial is completed.

The details of the methodology are sufficient to allow the study to be reproduced (with a few exceptions, listed above).

The manuscript is well organized and written clearly enough to be accessible to non-specialists?
