# Supplementary material for: Examining intentions to take iron supplements to inform a behavioral intervention: The Reduction in Anemia through Normative Innovations (RANI) project
Source: PLoS One. 2021 May 11;16(5):e0249646. doi: 10.1371/journal.pone.0249646 (PMC8112683; doi:10.1371/journal.pone.0249646)
Supplement: Attachment — Submitted filename: Response to Reviewers.docx [file pone.0249646.s003.docx]

| **Response to Reviewers** | | |
| --- | --- | --- |
| **Reviewer #1** | **Response** | **Page, line #** |
| However, the conceptual framework could be strengthened by reference to other relevant theories, such as social identity theory, theory of planned behaviour, theory or normative conduct, to place the work in a wider context. | This is a great suggestion, and we have now situated this study in the larger social norms literature by including each of these theories. See the new “Conceptual background on social norms” section. | 6, line 52 |
| **Methods suggestions:** Line 100. Change future tense to past tense.  Line 119-121. The final sentence of the sampling method was not quite clear to me. I think it may describe some stratification by caste and tribe, but this was not clear because the word stratification is not used. Description of the caste and tribe variables, in particular how many categories there are, would help with clarity. | Done. Thank you.  We added the word “stratification,” described which castes and tribes are the most marginalized, and stated that there are many castes and tribes within the whole state of Odisha. | 9, line 122 |
| A power calculation is needed. This study describes the analysis of the baseline data from a RCT. I assume the authors did a sample size calculation for the RCT, which would not be appropriate to report here. However, I would like to see some reassurance that the size of the sample is sufficient for the analysis that was undertaken. | Thank you for this comment. We added the following text to the methods section:  “The RCT from which these data are drawn was powered to detect changes in hemoglobin levels. We did not conduct a post-hoc power analysis for the current study given the limitations of this approach (Hoenig and Heisey, 2001), but note that this study has a large sample relative to the literature on behavioral intentions.” | 10 lines 136-139 |
| Line 164-166. The start of this sentence indicates that the questions quoted later in the sentence are all Yes/No questions, but the first one requires a number as the answer – this just needs re-phrasing. | We’ve clarified how this is measured with the following text: “Respondents were asked to assess the strength of perceived injunctive norms from three reference groups using the following three questions” | 12, lines 183-184 |
| The collective norms variable was created from responses to the Intention to take iron question. The section on collective norms (lines 173-179) is difficult to understand without the reader having prior knowledge of the survey question about Intentions. This could be resolved by moving the section on Intentions (line 211-221) to become the first variable in the list. | We’ve moved intentions to take IFA up so that it comes before collective norms. | 11, line 166 |
| Line 194-198. Briefly explain the rationale for including a question about pregnancy, when non3 of the women were pregnant. | We agree with the reviewer and we dropped the item that referred to pregnant women. We only have one item that makes up outcome expectations in this version and it refers to outcome expectations for non-pregnant women only. | 13, lines 213-217 |
| Line 218-221. I am not quite clear from this description what transformation was done. Did the authors try out a number of different options and find that squaring it was the most suitable approach? Or did they do something else and then square that? The Stata commands gladder and ladder can be helpful in selecting a suitable transformation. I am not suggesting the authors should use those commands, simply that it be explained more clearly. | We agree that this was not clear and we decided not to square the outcome variable. OLS regression is quite robust to violations of the normality of residuals assumption, particularly in samples as large as ours. Given this, we thought it was easier to interpret without a squared outcome variable. You will also see that we edited the tables and text to reflect this change. None of the major findings change as a result. | 11, 166-173 |
| Pearson’s correlations are always zero-ordered, i.e. they assess the level of correlation between two variables without reference to other variables. I don’t think it is necessary to include the term zero-ordered, and that term may confuse people who are not used to seeing it. | Removed. |  |
| Missing data - What proportion of the women who were identified for the study consented to join it? The amount of missing data appears to be very low. Out of 3914 people in the sample, 3793 were included in the analysis. I think this warrants mention – what was done to achieve such high responses? | We’ve added this information to the methods section.  “93% of women who were eligible, consented to join the study; only 47 refused to participate and 271 were not home. Our data collection partners returned to each house up to three times to achieve such a high response rate.” | 10, lines 243-245 |
| **Suggestions for presenting results:**  Table 1. It would be informative to include the collective norms measure in the table and the associated text.  Table 2. The table includes the correlations between all of the variables in the model, but only the correlations with the outcome are reported in the text. I think it would be useful to briefly summarise the associations between the other variables, i.e. reassuringly, there are no high levels of correlation that might be worrying.  Table 3. Add standard errors and confidence intervals for all variables. Make clear in the table whether the coefficients are standardised beta or not. I saw something in the text which suggested these were standardised, but I am not sure looking at this whether they are. The benefit of using the original scales is that the coefficients can be more easily interpreted, but standardised results offer the opportunity to compare between the variables. If the journal allows, you could add the standardised results as a supplementary file. | Table 1. Thank you for this suggestion. We would like to keep Table 1 as a basic descriptive table with demographic variables. We do describe collective norms in the text but since it uses the non-self mean approach, it is difficult to interpret as a number on its own.  Table 2. We’ve briefly summarized some of the other correlations in Table 2 in the methods section write up.  Table 3. We’ve added confidence intervals for all variables. We made it clear in Table 3 that all variables are standardized. We also noted this in the manuscript text. |  |
| **Discussion:**  Injunctive norms are operationalised in the survey as ‘influential referent groups’ in the women’s local communities (women, husbands, mothers-in-law, mothers). In the discussion (line 326-339) the authors suggest that informing women and their families that the Indian government recommends iron would lead to women believing they should take it. It seems rather a leap from finding that women are influenced by the expectations of their local community and family to suggesting that they will be influence by the expectations of the government. These are injunctive norms operating at two different levels and I don’t think the second level was asked about in the survey. It may be that the authors think that a government recommendation would influence the husbands, mother-in-law and mothers, or that the recommendation will work in some other way, but I didn’t find that clear in the paper. | This is a good point. The effect of the government information campaign depends in part on convincing the referent groups to adopt this norm and then they will have an impact on the intentions through perceived injunctive norms. We revised the text to the following,  “Given the importance of perceived injunctive norms from husbands in particular and also mother’s-in-law, and community members, efforts to communicate national recommendations that non-pregnant should take IFA are likely to be more effective if they target these audiences in addition to women themselves.” | 19, lines 549-546 |
| **Conclusion:**  If I am reading Table 3 correctly (if the results are standardised) the variable with the largest effect was self-efficacy. This is mentioned in the text, but I think it would also be useful to mention this in the Conclusion and also in the Abstract. It seems to suggest another possible intervention that would be worth pursuing in this context. | We mentioned the large of association between self-efficacy and intentions in both the abstract (results and conclusion) and again in the conclusion within the paper.  “Findings: Other significant factors included age, education, breastfeeding, knowledge, self-efficacy, and outcome expectations.”  “Conclusion: In this context, where the actual behavior is low but intentions to enact the behavior are high, starting an intervention with injunctive norms messaging (expectations around the behavior) and self-efficacy to enact the behavior is a logical first step.”  “Conclusion: Overall, our results indicate that shifting social norms within communities and improving people’s knowledge are not enough to drive behavior if individuals do not believe that they can engage in that behavior (self-efficacy) and that the behavior will result in desired outcomes (outcome expectations).” |  |
| **Reviewer #2** |  |  |
| 1. In general: Worth mentioning why the rate of anaemia in women In India is so high? Is it unusually high compared to other countries? If yes, why? | We’ve added a sentence explaining why anemia rates are so high in India and Southeast Asia.  “High rates of anemia in India and other parts of Southeast Asia reflect underlying social determinants such as poverty which results in diets low in iron and high levels of malaria, and other infectious diseases.” | 4, lines 19-21 |
| 2. Page numbers are always p10 | Thank you. |  |
| 3. L 14. Maybe revise sentence. It still sounds like pregnant women have more of a right not to be anaemic compared to non-pregnant women. I would recommend a formulation like “Both, pregnant and noon-pregnant women are equally…” | Thank you for this suggestion. We hope this revised text is clearer.  “While the intergenerational nature of anemia via pregnancy is critical, women’s health by itself, apart from her children or her role as mother, is also important. Both pregnant and non-pregnant women deserve to be healthy and anemia free.” | 4, lines 11-13 |
| 4. L 33 guidelines stating that… | Added the word, “stating.” Thank you |  |
| 5. L 34 ‘evidence’ to practice gap? Is this the right term here? I understand the problem is that the government does not practice what they preach? Not sure if evidence is the correct word | We’ve removed this phrase and streamlined the sentence to make this point clearer. | 5, lines 49-53 |
| 6. L 47-53 explain why norms are the main focus as compared to the other variables | We state that recent qualitative research is showing that norms are a promising approach to increase IFA use. | 6, lines 73-75 |
| 7. L 54-56 very nice part, gap is explained very well | Thank you. |  |
| 8. L 74 maybe add Schultz 2007 reference on inj and desc norms | Added. | 7, line 113 |
| 9. L 91 IFA intentions? Rethink terminology. IFA-taking intentions? Also, stick to one terminology for intentions throughout your manuscript. They are changing slightly throughout. | We’ve stuck to “intentions to take IFA” or “intentions” for short throughout the paper. |  |
| 10. L 102 onwards. Check numbers and written-out numbers. Sometimes you say 22%, sometimes twenty two percent. | We only use the number followed by % throughout. |  |
| 11. L 134 term “data collector”. Local enumerators? Research Assistants? Mention if they received previous training as well please | We used the term research assistants and mentioned their two-week training. | 10, line 239 |
| 12. L 145 Why was mobile phone ownership added as demographic variable? | We used mobile phone ownership as one proxy for socio-economic status and included this statement in the revised version. |  |
| 13. L 161 Did you look at individual inj norms? Was there one norm that was especially strong? E.g. the husband inj norm explained most variance? That would be interesting as well for the communication aspect | This is a great suggestion. Thank you for drawing this to our attention. We examined each individual injunctive norm message and found that all were significantly associated with intentions to take IFA but that husbands expectations had the greatest association. We’ve added this to the results and discussion section. | 10, line 254 |
| 14. L 197 You talk about during pregnancy suddenly whilst the whole study is about non-pregnant women? | We agree with the reviewer and we removed the one item referring to pregnant women. Therefore, the variable outcome expectations only has one item. We edited the text and tables to reflect this change. | 13, lines 328-331 |
| 15. L 205 well done pre-testing your items. Why did they only perceive this difficulty in this item? Was the Likert scale appropriate for all other items? | Overall, we found in our formative research that women’s risk perception for anemia was extremely low and they mostly perceived it as an illness during pregnancy. Therefore, it was difficult for non-pregnant women to assess risk for themselves in the span of one year. The likert scale worked well for other survey items. |  |
| 16. L 211 I have difficulties with the way you operationalized intentions. Why is there no basic intention to take IFA? Each intentions has a second part to it that says “even if” which is a mix of norms, self-efficacy and intentions. I think this needs to be discussed in the limitations and explained why this was needed. In my eyes, you cover self-efficacy and norms very well already and a basic three-item measure of intentions would suffice. Of course you can’t change this in retrospect but maybe discuss shortly and explain why this kind of intentions. | We’ve added this to the limitations section.  “We also chose to operationalize intentions based on barriers that we found in our formative research (e.g., taking it even if you are not pregnant and taking it even if your husband or mother-in-law does not want you to). While these questions illuminated specific sides of intentions, a simple question that did not take into account these barriers may have produced different results.” | 23, lines 649-653 |
| 17. L 290-301 onwards: maybe a bit more detail about what the stage model means and what kind of intervention fits your identified stage (inspiration from Bamberg 2013) | Thank you for this suggestion. This is an important addition. We’ve added specifics around what our findings may mean for an intervention and which stages we plan to start with. | 18, lines 508 - 517 |
| 18. L 302-310 it could also mean that actually, Descriptive norms have a lot of power influencing actual behaviour (as compared to intentions, because not many take IFA) but that it is not an explicit influence of norms as demonstrated in Nolan, Schultz et al 2008. | This is a good point. We will test whether or not descriptive norms has an effect on actual behavior at midline data collection when more women are actually taking IFA. We’ve added this into the text. | 18, lines 526-528 |
| 19. L321 what does improve social norms mean? | We revised this sentence to state, “Interventions may want to change social norms (e.g., increase perceived injunctive norms that non-pregnant women should be taking IFA) as well as these key moderators (that, in turn, augment the influence of norms).” | 19, lines 537-541 |
| 20. L 328 deviation in descriptive social norms was larger… | Edited. |  |
| 21. L 340-346 I really like this paragraph. | Thank you. |  |
| 22. L 353 increasing knowledge contradicts your Stage model claim? | We revised this to state that improving knowledge will be included in the intervention but not the focus due to the fact that women already have high intentions to take IFA. | 20, lines 575-592 |
| 23. I miss more specific ideas in the discussion about how to operationalize an intervention, what has been tested before to increase self-efficacy in a similar case for example and how would you do it? | This is also an important addition. We’ve added in specific suggestions about how to address barriers, change social norms, and increase self-efficacy to take IFA in an intervention. | 21, lines 608 - 627 |
| 24. L 369 Limitations. Did all people find it easy answering the scales? I find it sometimes hard to conduct survey studies with people from various background, which means facilitators have to engage more or less with some people which could lead to biases? | We pilot tested all of the questions and revised them to make them as simple to answer as possible. We also had daily debriefings after most data collection days. We agree that it is difficult to create a survey that is appropriate for participants from diverse backgrounds. Research assistants reported that participants found some of the questions redundant. To prevent fatigue, they took short breaks. |  |
| 25. L 392 – 395 very nice section | Thank you |  |
| 26. References. Make sure you add DOIs | Added. |  |
