# Supplementary material for: Examining intentions to take iron supplements to inform a behavioral intervention: The Reduction in Anemia through Normative Innovations (RANI) project
Source: PLoS One. 2021 May 11;16(5):e0249646. doi: 10.1371/journal.pone.0249646 (PMC8112683; doi:10.1371/journal.pone.0249646)
Supplement: Attachment — Submitted filename: Response to Reviewers.docx [file pone.0249646.s004.docx]

Dear Reviewers,

Thank you for suggesting additional ways to improve our manuscript. We’ve incorporated each suggestion in the manuscript and documented how we did it below.

| Reviewer #1: In response to my previous comments the authors have amended the Methods section to make it clear that stratification ‘by the proportion of minority populations’ and 20 clusters were selected from each strata. It would be informative to specify how the strata were categorised, for example, were the proportions of minority populations stratified into x groups according to some cut-points and then y clusters chosen from each strata? (page 9, line 22). | We added additional information on how the strata were categorized and chosen. Page 9, lines 134-139. |
| --- | --- |
| 1. Abstract background: replace “ignored” with “disregarded” (sounds less intentional) 2. Abstract methods spell out “other factors” (name these factors) | Edited.  Added. |
| 3. Abstract results: you cross out education, did you exclude it now? Did you include it before? Mention in the paper that you measured but excluded it from the analysis then. | Education was still included in the analysis but after making suggested methodological edits, education was only significant in the first model, not the second when we added in additional factors. Therefore, we removed it from the reported significant variables in the abstract. However, the more detailed results and tables highlight this nuance. |
| 4. Abstract conclusion: “logical first step” replace with “the step we recommend based on our results” | Edited. |
| 5. L 5: change order of sentence “a 50% reduction… is the second global target by the WHA” | Edited. |
| 6. L 16: replace deserve with shall be supported | Edited. |
| 7. L 22: poverty is often related to education as well, so definitely needs to be explained why you took it out of the model (would be a relevant predictor). Is Malaysia and infectious diseases related to iron deficiency as well? | As mentioned earlier, we did not take education out of the model. It was just no longer significantly associated with intentions to take iron supplements so we removed it from the significant results. |
| 8. L 29 change location of “weekly” back to where it was | Changed back. |
| 9. L 36 ff: Divide into 2 sentences to make clear | Divided. |
| 10. L 52: delete “to use” | Deleted. |
| 11. P 6 and 7 very nice changes and additions | Thank you. |
| 12. L 112 reformulate “at different times” over time? Prone to change? | Thank you for the suggestions but we want to state that descriptive and injunctive norms can change at different times so it’s important to measure them separately. |
| 13. L 152-164 very nicely explained | Thank you. |
| 14. L 178 consent for what? | Added. |
| 15. L 296: You indicate education level here? Did you exclude it or not? | We did not exclude it. |
| 16. L 300 ff and 381 ff: thought: is there any information or research about father’s husbands attitude to iron supplements? It seems important for women that the supplements are approved by their male household members, so maybe the campaign needs to target them in the first place? Do we know how they think about it? Do they support their daughters/wives or are they sceptical? I recommend going a little bit deeper into this aspect, also in the introduction and the implications in the end. | We feel that we’ve already mentioned that husbands are an important referent group, how husbands influence women more than other women, or mothers-in-law, and we suggest including them in the intervention. Here are the four places where we mention them.  “We chose husbands and mothers-in-law as the referent groups because of our formative work that highlighted their importance (Sedlander et al., 2020 Sedlander et al., 2018).”  “We found that they were all significantly associated with IFA intentions but the association was strongest when husbands expected their wives to take IFA, (β = .08, *p* < .001) compared to mothers-in-law (β = .06, *p* < .001), and other women in the community (β = .06, *p* < .001).”  “Raising awareness about anemia (to improve issue familiarity), highlighting the fact that taking IFA is easy (to enhance self-efficacy), that doing so is beneficial (outcome expectations), and asking husbands to ensure their wives are taking IFA (to enhance monitoring) are strategies that interventions could adopt.”  “Given the importance of perceived injunctive norms from husbands in particular and also mother’s-in-law, and community members, efforts to communicate national recommendations that non-pregnant should take IFA are likely to be more effective if they target these audiences in addition to women themselves.” |
| 17. The discussion improved a lot. | Thank you. |
| 18. L 521 ff: lovely finish | Thank you. |
